# Hippocampus Metabolic Disturbance and Autophagy Deficiency in Olfactory Bulbectomized Rats and the Modulatory Effect of Fluoxetine

**DOI:** 10.3390/ijms20174282

**Published:** 2019-09-01

**Authors:** Yunfeng Zhou, Xue Tao, Zhi Wang, Li Feng, Lisha Wang, Xinmin Liu, Ruile Pan, Yonghong Liao, Qi Chang

**Affiliations:** 1 Institute of Medicinal Plant Development, Chinese Academy of Medical Sciences and Peking Union Medical College, Beijing 100193, China; 2 School of Medicine, the Open University of China, Beijing 100039, China

**Keywords:** olfactory bulbectomy, depression, hippocampus, metabonomics, autophagy

## Abstract

An olfactory bulbectomy (OBX) rodent is a widely-used model for depression (especially for agitated depression). The present study aims to investigate the hippocampus metabolic profile and autophagy-related pathways in OBX rats and to explore the modulatory roles of fluoxetine. OBX rats were given a 30-day fluoxetine treatment after post-surgery rehabilitation, and then behavioral changes were evaluated. Subsequently, the hippocampus was harvested for metabonomics analysis and Western blot detection. As a result, OBX rats exhibited a significantly increased hyperemotionality score and declined spatial memory ability. Fluoxetine reduced the hyperemotional response, but failed to restore the memory deficit in OBX rats. Sixteen metabolites were identified as potential biomarkers for the OBX model including six that were rectified by fluoxetine. Disturbed pathways were involved in amino acid metabolism, fatty acid metabolism, purine metabolism, and energy metabolism. In addition, autophagy was markedly inhibited in the hippocampus of OBX rats. Fluoxetine could promote autophagy by up-regulating the expression of LC3 II, beclin1, and p-AMPK/AMPK, and down-regulating the levels of p62, p-Akt/Akt, p-mTOR/mTOR, and p-ULK1/ULK1. Our findings indicated that OBX caused marked abnormalities in hippocampus metabolites and autophagy, and fluoxetine could partly redress the metabolic disturbance and enhance autophagy to reverse the depressive-like behavior, but not the memory deficits in OBX rats.

## 1. Introduction

Major depressive disorder (MDD) is a serious mental disorder with a high risk of suicide and a lifetime prevalence of 15% to 18%, which severely diminishes the life quality of patients and causes heavy financial burden for society [1]. As a multifactorial disorder, the pathophysiology of MDD is complicated and still not clear. Animal models are crucial tools to investigate the precise molecular mechanism of MDD [2]. Bilateral olfactory bulbectomy (OBX) causes a series of abnormal behavioral changes in rodents including hyperlocomotion, anhedonia, and hyperemotionality, which can mimic the clinical symptoms of patients with MDD, especially for agitated depression. Moreover, memory impairment, cholinergic deficiency, and accumulative Aβ have also been observed in OBX rodents [3,4], which implies the validity of this model in cognitive disorders. At present, the OBX model has been widely applied to explore the molecular mechanism of MDD and Alzheimer’s disease (AD), and evaluate the corresponding therapeutic drugs [5,6].

Recently developed metabonomics technology has become a powerful approach in the exploration of pathological processes, the identification of potential biomarkers, and the explanation of biochemical pathways in diverse complex diseases [2], including depression and AD. The urinary metabolic profile in OBX rats has been investigated by the metabonomics approach in our recent report [7]. Hippocampus is a critical brain region in the regulation of memory and mood, and hippocampus metabolic profiles have been explored in various depression-related and AD-related animal models by using the metabonomics strategy. The disturbances in amino acid, fatty acid, and phospholipid metabolism have been reported in the hippocampus of chronic unpredictable mild stress (CUMS)-induced rats [8,9]. Additionally, disrupted carbohydrate metabolism and vitamin metabolism were discovered in the hippocampus of APPswe/PS1dE9 mice, which is a transgenic model for AD [10]. Although the dysfunction of various cellular processes within the hippocampus of OBX rats has been discussed in detail by a number of studies [11], alterations in hippocampus metabolites are still unknown in this model.

Autophagy is an intracellular degradation process, in which a part of the cytoplasm was surrounded by an isolated membrane to form a double-membrane vesicle, which is known as autophagosome (AP). The proteins and cellular structures in AP were degraded by the fusion of AP with lysosomes [12]. Autophagy is a conserved catabolic event and plays an indispensable role in eliminating impaired organelles and misfolded proteins [13]. Recent research studies showed that autophagy deficiency disrupted cellular energy homeostasis in the central nervous system, and closely associated with depression and neurodegenerative disorder [14,15,16]. The mammalian target of rapamycin (mTOR) is a key energy sensor in regulating the balance between cell growth and autophagy in response to the nutritional status. The accumulating evidence displayed that both amino acids and glucose could regulate the activity of mTORC1 (mTOR complex 1, comprised of mTOR, mLST8, PRAS40, and raptor) to make a difference in autophagy [17]. Furthermore, an increased AMP/ATP ratio promoted the phosphorylation of AMP-activated protein kinase (AMPK), which further inhibited the activity of mTORC1 [17].

mTOR served as a negative regulator in autophagy, and the inhibitor of mTOR, known as rapamycin, exerted anti-depressive-like effects in rodents after sub-chronic treatment [18], which suggests that enhanced autophagy might contribute to the therapy of depression. It has been well demonstrated that, beyond the effects on monoaminergic neurotransmitters, some antidepressants could facilitate autophagy during the process of relieving depressive symptoms. For example, paroxetine and amitriptyline decreased the immobility in a forced swimming test (FST) in mice and increased the levels of beclin1, autophagy related protein 12 (ATG 12), and LC3B II/LC3B I in the prefrontal cortex (PFC) and hippocampus [19]. Furthermore, fluoxetine and paroxetine enhanced the mRNA level of LC3 and beclin1 in blood mononuclear cells in depressed patients [20]. Up to now, the autophagy status has never been investigated in the hippocampus of OBX rats and the impact of fluoxetine on autophagy is also far from clear.

Given the considerations above, ultra-performance liquid chromatography quadrupole the time of flight mass spectrometry (UPLC-QTOF-MS)-based metabonomics, which was employed to reveal hippocampus metabolic changes in OBX rats, and, meanwhile, the regulation effects of fluoxetine on behaviors, hippocampus metabolites, and proteins associated with autophagy were also evaluated.

## 2. Results

### 2.1. Behavioral Changes in Rats Subjected to OBX and Fluoxetine Treatment

**Figure 1:**
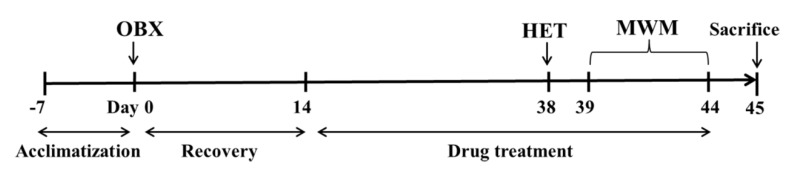
Diagram of the experimental protocol. HET, Hyperemotionality Test. MWM, Morris Water Maze. OBX, Olfactory Bulbectomy.

**Figure 2:**
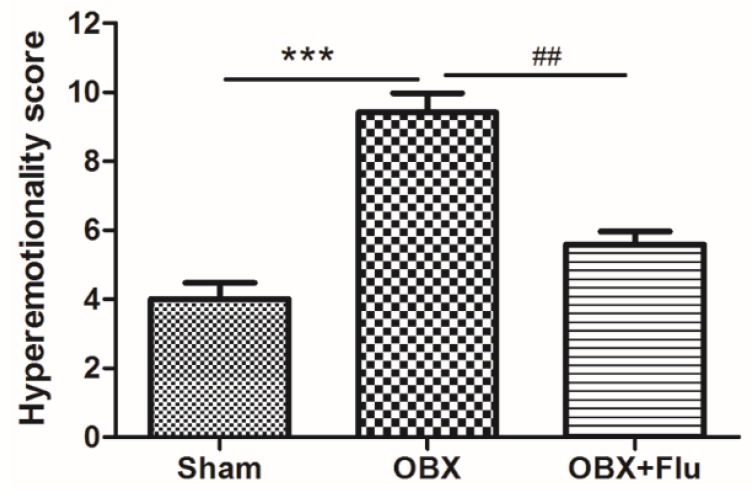
The effects of OBX and fluoxetine treatment on the hyperemotionality score in rats. *** *p* < 0.001 compared to the sham group. ## *p* < 0.01 compared to the OBX group (means ± SEM, *n* = 8). Flu, fluoxetine. OBX, olfactory bulbectomy.

The detailed experimental schedule was presented in Figure 1. Hyperemotionality is one of the most typical behavior features of OBX rats, which simulates the depressed patients with psychomotor agitation. OBX rats exhibited intense irritability, including the increased attack, struggle, startle, and fight responses. The one-way ANOVA test of the results in the hyperemotionality test revealed a significant difference among the three groups (F_(2,21)_ = 20.25, *p* < 0.001). As shown in Figure 2, the hyperemotionality score was remarkably higher in OBX rats than in sham rats (*p* < 0.001). Chronic fluoxetine treatment reversed the abnormal behaviors in the hyperemotionality test (*p* < 0.01).

**Figure 3:**
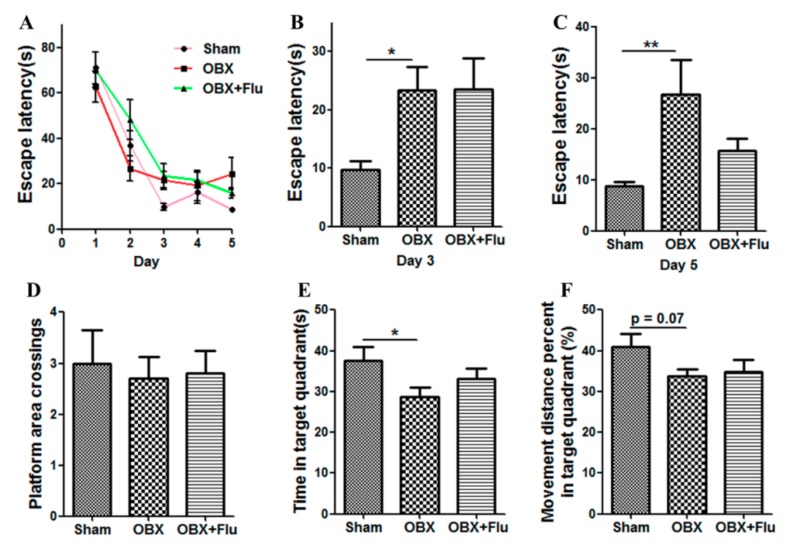
Effects of OBX and fluoxetine treatment on spatial memory of rats in the Morris water maze (MWM) test. (**A**) The latency to the hidden platform during the five-day navigation test. (**B**,**C**) The escape latency recorded on day 3 and day 5, respectively. On day 6, the platform was withdrawn. (**D**) Platform area crossings. (**E**) Time spent in the target quadrant. (**F**) Movement distance percent in the target quadrant. * *p* < 0.05, ** *p* < 0.01 compared to the sham group (means ± SEM, *n* = 8). Flu, fluoxetine. OBX, olfactory bulbectomy.

The Morris water maze (MWM) test is a classical approach to estimate the spatial memory in rodents. There are significant differences in the escape latency on day 3 (F_(2,21)_ = 3.73, *p* < 0.05) and day 5 (F_(2,21)_ = 4.74, *p* < 0.05) among the three group by one-way ANOVA. Further post-hoc analysis showed that OBX caused severe impairment in learning and memory in rats, which is displayed by significantly increased escape latency on day 3 and day 5 (*p* < 0.05 and *p* < 0.01, respectively) in OBX rats compared to sham rats in the navigation test (Figure 3A–C). In the probe test, there are no significant differences in the number of platform crossings (F_(2,21)_ = 0.09, *p* = 0.91, Figure 3D), the time spent in the target quadrant (F_(2,21)_ = 2.79, *p* = 0.08, Figure 3E), and the movement distance percent in the target quadrant (F_(2,21)_ = 1.51, *p* = 0.24, Figure 3F) among the three groups. However, OBX caused a marked decrease in the time spent in the target quadrant (*p* < 0.05) and a decline trend in the movement distance percent in the target quadrant (*p* = 0.07) in rats. Fluoxetine treatment could not alleviate the memory deficits in OBX rats in the MWM test.

### 2.2. UPLC-QTOF-MS Method Validation

Excellent reproducibility is essential for sample analysis in untargeted metabonomics. In the present research, quality control (QC) samples were used to estimate the repeatability, precision, and stability. Assay repeatability and instrumental precision were estimated by detecting 6 parallelly prepared QC samples and the same QC sample for 6 replicates, respectively. The sample stability was measured by analyzing the same QC sample (kept at 4 ^°^C) at 0, 8, 16 and 24 h after preparation. Twelve ion chromatographic peaks (retention time (min)_m/z: 0.90_102.05996, 2.78_137.047, 3.20_120.0818, 5.01_621.0631, 6.97_714.7231, 8.26_820.6949 in a positive ionization mode and 0.89_329.0179, 3.52_218.1045, 5.22_255.0907, 8.31_242.1789, 10.39_541.3306, 12.41_259.2411 in a negative ionization mode) in hippocampus QC samples were extracted to verify method reproducibility. The results showed that relative standard deviation (RSD) values of peak areas and retention times for 12 extracted peaks were 1.76–13.88% and 0.00–2.35% (Table S1), which suggests that the current method is reliable for the analysis of metabolites in hippocampus samples.

### 2.3. Hippocampus Metabolic Profile Analysis

**Figure 4:**
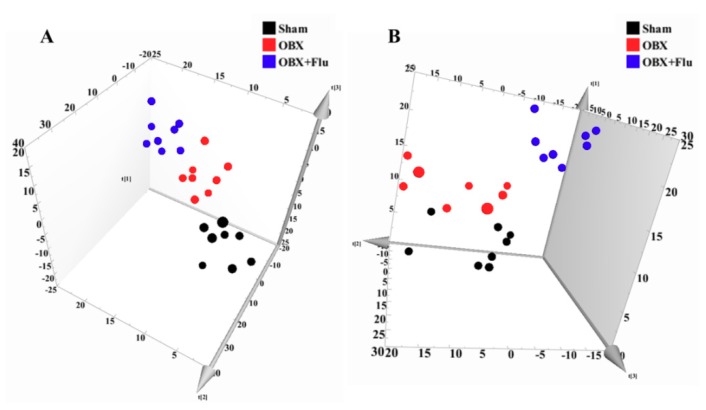
3D-PCA score plots of hippocampus samples from sham (black), OBX (red), and OBX + Flu groups (blue) assayed by the UPLC-QTOF-MS in positive (**A**) and negative (**B**) ionization modes, respectively (*n* = 8). Flu, fluoxetine. OBX, olfactory bulbectomy.

**Figure 5:**
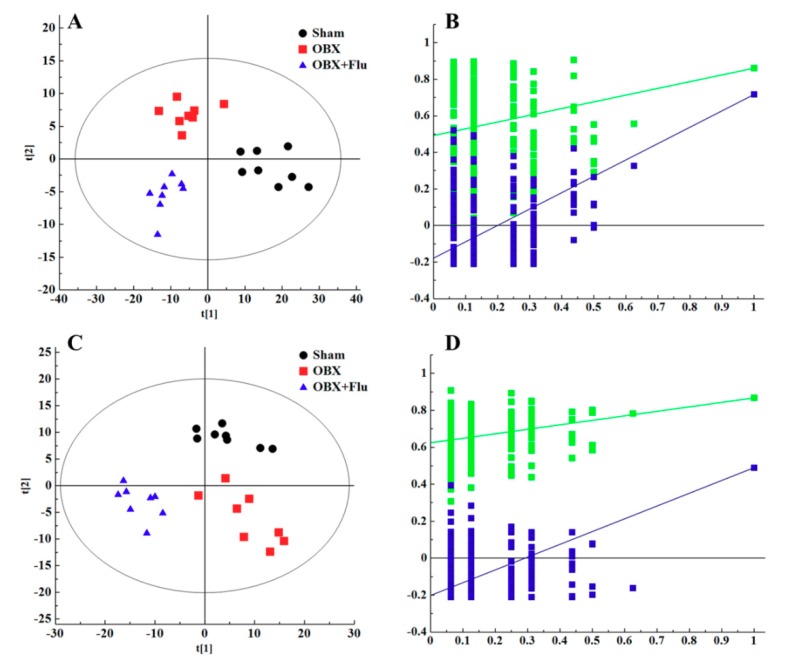
PLS-DA score plots of hippocampus samples from sham group (black), OBX group (red), and OBX + Flu group (blue) in a positive ionization mode (**A**), (R2X = 0.281, R2Y = 0.853, Q2 (cum) = 0.484) and negative ionization mode (**C**), (R2X = 0.236, R2Y = 0.805, Q2 (cum) = 0.516) (n = 8). The results of 200 permutations in PLS-DA models showed that R2 (green square) and Q2 (blue square) values in the intercept of the Y-axis (bottom left) were significantly lower than their corresponding original R2 and Q2 values (top right) in a positive ionization mode (**B**), (R2 = 0.492, Q2 = −0.178) and a negative ionization mode (**D**), (R2 = 0.625, Q2 = −0.202), respectively. Flu, fluoxetine. OBX, olfactory bulbectomy.

In multivariate data analysis, principal component analysis (PCA) was first performed to depict the general tendency of samples. The samples in sham, OBX and OBX + Fluoxetine groups, were clearly separated in the three-dimensional (3D)-PCA score plot in both positive and negative ionization modes (Figure 4A,B), which indicated that remarkable metabolite changes occurred in the hippocampus after rats were subjected to OBX and fluoxetine treatment. Partial least squares discriminant analysis (PLS-DA) was used to find the differential metabolites and a clearer separation among these three groups was observed in PLS-DA plots in both positive and negative ionization modes (Figure 5A,B). R2Y represented the explanatory validity and Q2 implied the predictive validity of the PLS-DA model. The values of R2Y and Q2 calculated by SIMCA-P (version 13.0) were 0.853 and 0.484 in the positive ionization mode and 0.805 and 0.516 in the negative ionization mode, respectively, which suggests that the PLS-DA model had high fitness ability and middle predictability. The intercepts of R2 and Q2 of 200-permutation test were 0.492 and −0.178 in a positive ionization mode (Figure 5B) and 0.625 and −0.202 in a negative ionization mode (Figure 5D). The hippocampus metabolites with the variable importance in the projection (VIP) > 1 in the PLS-DA model and *p* < 0.05 in the *t*-test were regarded as potential biomarkers.

### 2.4. Identification of Potential Biomarkers

**Figure 6:**
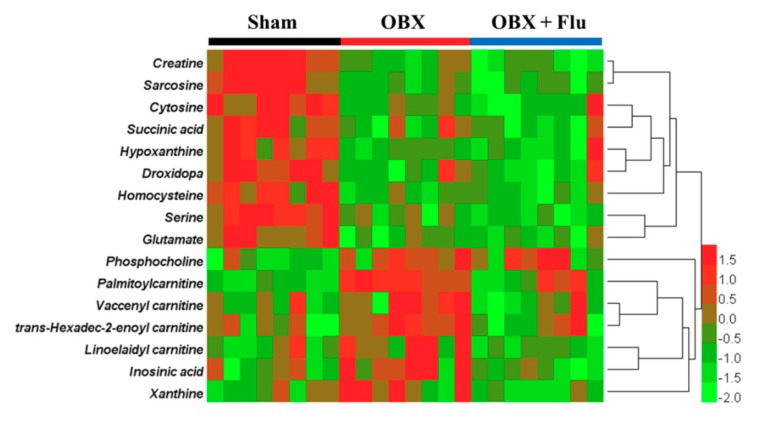
Heat map of identified biomarkers in the hippocampus of OBX rats. Red and green represent that the levels of potential biomarkers are higher and lower compared to the average level, respectively. Rows imply the potential biomarkers and columns represent hippocampus samples of rats (*n* = 8 in each group). Flu, fluoxetine. OBX, olfactory bulbectomy.

Human Metabolome Database (HMDB, http://www.hmdb.ca/) and MassBank database (http://www.massbank.jp/) were applied for metabolite identification. The exact molecular weights of metabolites were submitted to online databases, and possible chemical formulas with mass errors within 10 ppm were selected for further analysis. The final structures of the compounds were determined by comparing the ion fragments in raw data with these in databases. To take the biomarker with m/z 347.0355 in negative ionization mode as an example, the chemical formula was confirmed to be C_10_H_13_N_4_O_8_P by HMDB. Additionally, fragment ions at m/z 304.0287, 177.0473, 135.0322, and 78.9598 in MS/MS spectra represented [M-CHNO-H]^−^, [M-C_3_H_7_O_6_P-H]^−^, [M-C_5_H_9_O_7_P-H]^−^, and [M-C_10_H_12_N_4_O_5_-H]^−^, respectively. Therefore, the compound was identified as inosinic acid by the MS/MS information in the database and raw data. As a consequence, a total of 16 metabolites in the hippocampus were identified as potential biomarkers for OBX-induced model of depression and neurodegeneration (listed in Table 1). Fluoxetine treatment corrected the levels of trans-hexadec-2-enoyl carnitine, palmitoylcarnitine, linoelaidyl carnitine, vaccenyl carnitine, xanthine, and inosinic acid, but aggravated the concentrations of serine and creatine. The variation trends of candidate biomarkers among the three groups were visualized by a heat map using HemI software (version 1.0.3.3) [21] (Figure 6). The affected pathways recognized by the KEGG Pathway Database (https://www.kegg.jp/kegg/pathway.html) were involved in amino acid metabolism, fatty acid metabolism, purine metabolism, and energy metabolism.

### 2.5. The Changes in the Expression of Protein Associated to Autophagy in OBX and the Roles of Fluoxetine

**Figure 7:**
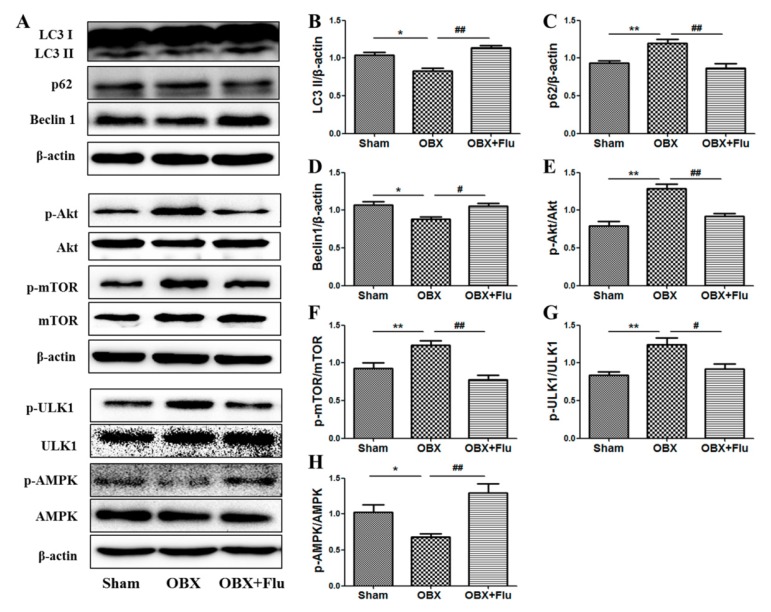
Effects of OBX and fluoxetine treatment on the expression of proteins associated with autophagy in the hippocampus of rats. (**A**) Representative protein bands. (**B**–**H**) The relative grey values of LC3 II/β-actin, p62/β-actin, beclin1/β-actin, p-Akt/Akt, p-mTOR/mTOR, p-ULK1/ULK1, and p-AMPK/AMPK. * *p* < 0.05, ** *p* < 0.01 compared to the sham group. # *p* < 0.05, ## *p* < 0.01 compared to the OBX group (mean ± SEM, *n* = 4). Flu, fluoxetine. OBX, olfactory bulbectomy.

LC3 II, p62, and beclin1 were detected in the hippocampus to measure the alterations in autophagy. The one-way ANOVA test showed a significant difference in the levels of LC3 II (F_(2,9)_ = 15.70, *p* < 0.01, Figure 7B), p62 (F_(2,9)_ = 8.85, *p* < 0.01, Figure 7C), and beclin1 (F_(2,9)_ = 6.71, *p* < 0.05, Figure 7D) among the three groups. Compared to sham rats, significantly decreased expression of LC3 II and beclin1 (*p* < 0.05), and markedly increased level of p62 (*p* < 0.01) in the hippocampus indicated autophagy was suppressed in OBX rats. Fluoxetine treatment (10 mg/kg, per os (p.o.)) remarkably up-regulated the expression of LC3 II and beclin1 (*p* < 0.01 and *p* < 0.05, respectively), and down-regulated the level of p62 (*p* < 0.01), which suggests that fluoxetine could improve autophagy in OBX rats.

**Figure 8:**
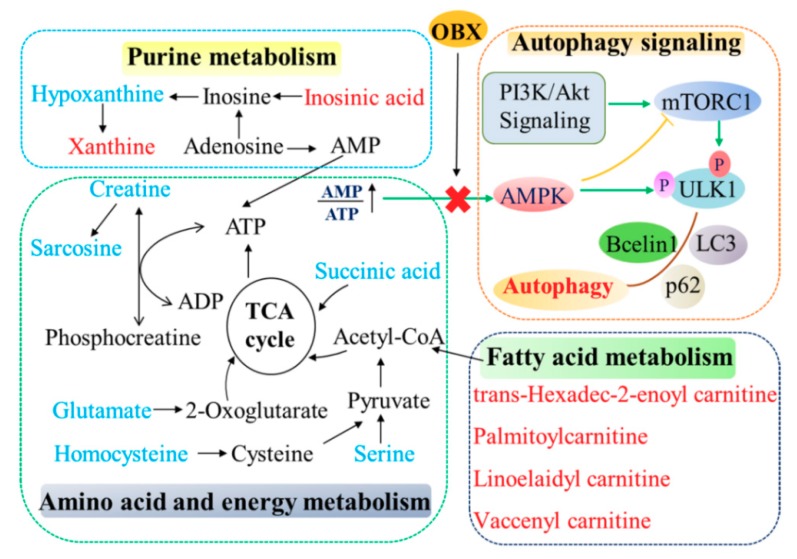
The correlation network of altered metabolites and autophagy in the hippocampus of OBX rats. The metabolites marked in blue denote decreased biomarkers and those in red indicate the increased ones.

AMPK and mTOR play key roles in the regulation of autophagy. A one-way ANOVA test revealed that there are significant differences in the expression of p-Akt/Akt (F_(2,9)_ = 13.21, *p* < 0.01, Figure 7E), p-mTOR/mTOR (F_(2,9)_ = 9.15, *p* < 0.01, Figure 7F), p-ULK1/ULK1(F_(2,9)_ = 10.99, *p* < 0.01, Figure 7G), and p-AMPK/AMPK (F_(2,9)_ = 15.01, *p* < 0.01, Figure 7H). The level of p-mTOR/mTOR was significantly elevated in the hippocampus of OBX rats (*p* < 0.01), and similar results were observed in the expression of p-Akt/Akt (the upstream of mTOR) (*p* < 0.01) and p-ULK1/ULK1 (*p* < 0.01) (the downstream of mTOR). On the contrary, the ratio of p-AMPK/AMPK was significantly reduced in OBX rats compared to sham rats (*p* < 0.05,). Chronic administration of fluoxetine inhibited the activation of Akt, mTOR, and ULK1 and increased the phosphorylation level of AMPK.

(The correlation network of altered metabolites and autophagy in the hippocampus of OBX rats was exhibited in Figure 8.)

## 3. Discussion

Agitated depression is characterized by irritability, psychomotor agitation, and a high risk of suicide, accompanied with loss of appetite, and feelings of guilt and worthlessness [22]. The behavioral characteristics in OBX rats, such as hyperemotional behavior, hyperactivity, and the decrease in memory ability, make this model not only suitable for agitated depression, but also valid for AD. In the present study, OBX rats displayed a significantly increased hyperemotionality score when experiencing four mild stimuli. Similarly to previous studies [23,24], we observed that OBX rats exhibited significantly prolonged escape latencies on day 3 and day 5 in the MWM navigation test, and shortened time and movement distance percentage in the target quadrant in the probe test, which indicates the spatial memory deficits in OBX rats. Chronic fluoxetine treatment relieved the hyperemotional response, but could not ameliorate impairment in learning and memory in OBX rats. These findings are in agreement with a previous report [25], in which fluoxetine reversed the depressive-like behaviors, but fail to enhance the learning and memory ability in OBX mice in the MWM test.

The hippocampus is an essential brain region for various behavior and physiology processes, such as spatial navigation, the regulation of the hypothalamic pituitary adrenal axis, and emotional control [26,27]. The olfactory bulb can send projections to the hippocampus directly or indirectly (via the piriform cortex or entorhinal cortex). Therefore, it is inevitable that OBX leads to dramatic and persistent abnormal changes in the structure and function of the hippocampus. OBX induced the inhibition in cell proliferation and survival in dentate gyrus (DG) [28,29], decreased the long-term potentiation in DG and CA1 regions [30], destroyed synaptic plasticity [11], and induced neuroinflammation in the hippocampus [31,32]. However, metabolite changes in the hippocampus have never been reported in OBX animals. We found that metabolic profiles in the hippocampus changed markedly in rats suffering from OBX. Sixteen metabolites in the hippocampus were identified as potential biomarkers for OBX-induced depression. Among them, six metabolites were restored by fluoxetine treatment (10 mg/kg, p.o.) for 30 days. The disorganized pathways correlated to these endogenous biomarkers included amino acid metabolism, fatty acid metabolism, purine metabolism, and energy metabolism. Similar metabolic changes were also found in the urine of OBX rats in our previous research, in which 26 altered metabolites were regarded as urine biomarkers for the OBX model and 15 of them were normalized by fluoxetine treatment [7]. These results indicated that fluoxetine partly alleviated the turbulent metabolic pathways not only in the urine, but also in the hippocampus of OBX rats.

The disturbance in amino acid metabolism has been reported in plasma of patients with MDD [33], and several brain regions in learned helplessness-induced and CUMS-induced depressed rats [2,34,35]. In our study, the amino acids (glutamate, serine, sarcosine, and homocysteine) were consistently decreased in the hippocampus of OBX rats. Glutamate is a major excitatory neurotransmitter in the mammalian nervous system, which can activate the *N*-methyl-d-aspartate (NMDA) receptor and play an indispensable role in modulating mood, learning and memory, and neuroplasticity [36]. Therefore, a decreased glutamate level may lead to the damage in cellular and synaptic plasticity in the hippocampus of OBX rats [11]. Although serine was defined as a biomarker, it is difficult to confirm its configuration as d-isomers or l-isomers by UPLC-QTOF-MS. d-Serine, synthesized by the isomerization of l-serine in neurons and astrocytes [37], is a co-agonist of the NMDA receptor and has great influence on synaptic plasticity through glutamatergic system [38]. The release of d-serine can be triggered by glutamate [39], so a reduced glutamate level might lessen the release of serine in our present study. The antidepressant effects of acute and chronic d-serine or l-serine treatment have been investigated in serotonin depletion, learned helplessness, and the OBX paradigm [37,39,40]. Sarcosine, also as a co-agonist of NMDAR, could alleviate depressive symptoms in patients and reverse behavioral deficits in CUMS-induced animals, and its antidepressant effects were superior to citalopram [41]. These reports indicated that enhancing the NMDA receptor function may benefit the therapy of depression. Homocysteine is a nonessential amino acid derived from the metabolism of methionine, and a body of evidence implied that elevated plasma homocysteine was considered a risk factor for cognitive impairment and depression in older adults [42,43,44]. The differences in species (human or rodent) and samples for analysis (plasma or hippocampus) might explain the contrary results in homocysteine level between patients and OBX rats, although further study is needed.

The human brain accounts for only 2% of body weight, but consume around 20% of the energy. Therefore, sufficient ATP supply is necessary for the brain to maintain normal physiological function [45]. Several metabolites related to energy metabolism changed greatly in the hippocampus of OBX rats. Creatine, as an energy carrier, can receive an N-phosphoryl group from ATP to produce phosphocreatine under the catalysis of creatine kinase. Phosphocreatine can cross the cell membrane, located in the tissues as a lack of energy, and then release ATP and creatine by a creatine kinase isoenzyme [46]. Decreased levels of creatine were also confirmed in different brain regions in various animal models of depression [47,48,49]. However, the four acylcarnitine types (trans-Hexadec-2-enoyl carnitine, palmitoylcarnitine, linoelaidylcarnitine, and vaccenyl carnitine) involved in fatty acid metabolism were consistently up-regulated in the hippocampus of OBX rats. Acylcarnitines are fatty acid derivative esters of carnitine, which mediates the import of long-chain and medium-chain fatty acid from cytoplasm into mitochondria for beta-oxidation [50] and play essential roles in energy production. The increased levels of these metabolites might indicate the compensatory mechanism of energy production to resist negative effects of OBX. In addition, the concentration of palmitoylcarnitine had a clearly positive correlation with apoptosis [51]. Therefore, the elevation of palmitoylcarnitine may also be associated with the excessive apoptosis in the hippocampus CA1 region of OBX rats [52]. Purine not only plays a role in promoting neuronal differentiation, but also participates in energy transfer. The disruption in purine metabolism has been revealed in the patients with MDD and AD [53,54]. In the present study, elevated xanthine and inosinic acid and diminished hypoxanthine were observed in the hippocampus of OBX rats.

Acetyl-CoA is an important intermediate involved in the TCA cycle, and is mainly generated from glycolysis, fatty acid beta-oxidation, and catabolism of the amino acid [50]. The TCA cycle is the major approach to produce ATP for the human body. In our study, biomarkers involved in amino acids, fatty acids, and purine metabolisms had an impact on energy generation or transfer, which indicates the unbalanced energy status in the hippocampus of OBX rats. The levels of four acylcarnitines, xanthine, and inosinic acid could be corrected by fluoxetine treatment, which indicates that the antidepressant effects of fluoxetine can be, at least partly, attributed to its regulation effects in energy metabolism.

mTOR and AMPK are both the major sensors in cellular nutritional status and plays important roles in autophagy, which can be affected by fluctuated energy metabolism. mTOR is activated under the condition of energy sufficiency and exerts an inhibitory effect on autophagy. Under nutrient starvation, activity of mTOR is suppressed, which serves as an initial signal for autophagy [55]. AMPK was regarded as an energy-sensing kinase and is activated by ATP depletion [56]. Activated AMPK can further phosphorylate the tuberous sclerosis complex 2, which is a modulator of mTORC1, that inhibits mTOR. Therefore, AMPK is identified as a positive regulator of autophagy [57]. The elevated ratio of p-mTOR/mTOR and decreased level of p-AMPK/AMPK observed in the present study reflected the physiologic compensation in energy supply after rats that experienced OBX. Activated mTOR and lessened activity of AMPK were also revealed in the hippocampus and PFC of the CUMS-induced animal model [58,59], which suggests that different animal models of depression (surgery or stress) may share a common nutrient compensatory mode. Akt can positively regulate mTORC1 through a growth factor signaling. Similar to mTOR activation, the phosphorylation level of Akt was markedly enhanced in OBX rats, which is consistent with previous findings that OBX caused significant activation in PI3K-Akt-mTOR pathway in the hippocampus or PFC of rats [60,61]. Furthermore, mTOR can directly phosphorylates ULK1 at Ser757 to lower autophagy, proven by the increased expression of p-ULK1 (Ser757) in OBX rats. LC3 II (a credible autophagy marker for AP formation) and beclin1 (playing roles in the initial form of AP) was significantly decreased, and p62 (a cargo receptor in autophagy) was markedly elevated in OBX rats, which implies that OBX induced autophagy impairment in rats. Chronic administration of fluoxetine not only redressed the levels of LC3 II, beclin1, and p62, but also alleviated the abnormalities in AMPK and mTOR pathways in the hippocampus of OBX rats. Additionally, the positive impact of fluoxetine on autophagy in our research has also been verified in clinical patients with MDD [20]. (The correlation network of altered metabolites and autophagy in the hippocampus of OBX rats was exhibited in Figure 8.)

In summary, OBX caused remarkable alterations in the hippocampus metabolic profile of rats uncovered by UPLC-QTOF-MS-based metabonomics. Sixteen metabolites were identified as potential biomarkers in OBX rats, which is an animal model for depression and neurodegeneration. Moreover, autophagy in the hippocampus of rats was inhibited by OBX. Fluoxetine could correct some abnormal metabolites and enhance autophagy in the hippocampus to exert antidepressant effects, but with no effects on the memory deficit in OBX rats. The present study could facilitate our understanding on the pathological mechanism of depression from the perspectives of metabonomics and autophagy, and the antidepressant roles of fluoxetine.

## 4. Materials and Methods

### 4.1. Chemicals and Reagents

Fluoxetine·HCl (≥98%) was commercially provided by Aladdin Biochemical Technology Co. Ltd. (Shanghai, China). Acetonitrile (ACN) and formic acid (FA) in the MS grade were purchased from Thermo Fisher Scientific Co. Ltd. (Waltham, MA, USA). A Milli-Q Ultrapure water system (Boston, MA, USA) was used to produce ultrapure water.

### 4.2. Animals

Male Sprague-Dawley rats (220–240 g) were purchased from Beijing HFK Bioscience Co., LTD (Beijing, China). Animals were maintained under conditions of standard lighting (light on at 08:00–20:00), temperature (23 ± 2 °C), and humidity (55% ± 5%) with free access to food and filtered water. The animals were habituated to daily handling for a week before the experiment. All the animal experiments were conducted in agreement with the Provision and General Recommendation of Chinese Experimental Animals Administration Legislation and were approved by the Animal Ethics Committee in our institute (SLXD-20180912006, 12 September 2018).

### 4.3. OBX Surgery

After a seven-day adaption, rats were subjected to bilateral OBX according to the previously described methods [62,63]. Rats were anesthetized with 2% pentobarbital sodium (50 mg/kg, intraperitoneal injection (i.p.)) and fixed in stereotaxic apparatus. After the skull was exposed, two holes were drilled on either side of the midline (2 mm in diameter, 8 mm anterior to bregma, and 2 mm lateral to the midline). The removal of olfactory bulbs was performed by aspiration with a blunt syringe needle attached to a vacuum pump. The holes were filled with absorbable collagen sponge immediately to control the bleeding and then the wound was sutured. Sham-operated rats experienced the same procedures, but their bulbs were left intact. After surgery, the rat was housed in an individual cage and given penicillin sodium (10,000 U in normal saline, i.p.) once a day for three consecutive days to prevent probable infection.

### 4.4. Drug Treatment

Following a 14-day recovery period, the rats that suffered from OBX were divided into two groups randomly (i.e., OBX group and OBX + Flu group). The rats in the OBX + Flu group were treated orally with fluoxetine (10 mg/kg, p.o.) once daily for 30 days, and the rats in sham and OBX groups were given with the vehicle. The dose of fluoxetine was consistent with a previous report [63]. There were 8 rats in each group.

### 4.5. Hyperemotionality Test

The hyperemotionality test was carried out on the 39th day post-surgery in accordance with previously reported methods [64,65]. The following four stimuli were used to measure the hyperemotionality, i.e., (1) attack response, a rod 4–5 cm presented in front of the snout, (2) struggle response, handling the rats with a gloved hand, (3) startle response, air blowing onto the dorsum of rats using a 10-mL syringe, and (4) fight response, tail pinching with the forceps. These responses were graded by a trained observer who was blind to experiment design as follows: 0, no response, 1, slight response, 2, moderate response, 3, marked response, and 4, extreme response. During each test, vocalization was scored as follows: 0, no vocalization, 1, occasional vocalization, and 2, marked vocalization. The total scores were comprised of emotional response and vocalization scores.

### 4.6. MWM Test

The MWM test was performed according to the previously described methods to evaluate the spatial learning and memory of rats [66,67]. A circular tank (150 cm in diameter and 50 cm high with a black wall) was filled with opaque water (25 ± 2 °C), and the tank was divided into four virtual quadrants. A platform (10 cm in diameter and 25 cm high) was localized in the center of a target quadrant and submerged 1 cm beneath the surface of water. A tracking system was used to record the performance of rats. In navigation training, each rat was given two trials per day for five consecutive days, with an inter-trial interval of 40 min. A rat was placed into one of the three quadrants (except for the target quadrant) with its head toward the wall. If the rat located the platform within 90 s, it was allowed to return to the home cage. If the rat failed to find the hidden platform within 90 s, it was gently steered toward the platform and allowed to stay there for 10 s. On day 6 of the MWM task, a probe test was conducted to assess the reference memory of rats, during which the platform was withdrawn. The rat was placed into the site opposite of the target quadrant and was monitored for 90 s. The latency to the hidden platform was recorded in the navigation test, and the number of times to cross the platform, the time spent, and movement distance percentage in the target quadrant were recorded in the probe test.

### 4.7. Hippocampus Collection and Sample Preparation

After behavioral tests, the rats were anaesthetized with pentobarbital sodium (50 mg/kg, i.p.), and 100 mL cold normal saline was perfused via hearts. The hippocampus was harvested on ice, frozen in liquid nitrogen immediately, and then stored at −80 °C until analysis. The left hemisphere of the hippocampus was used for UPLC-QTOF-MS detection and the right hemisphere was applied for a Western blot assay.

For metabonomics research, the hippocampus was homogenized in prechilled acetonitrile-water (1:1, *v*/*v*) with 10 volumes on ice. After centrifugation at 13,000 rpm for 15 min at 4 °C, the supernatant was collected and dried by a CentriVap vacuum concentrator (LABCONCO, MO, USA). The residue was re-dissolved with 100 μL acetonitrile-water (1:1, *v*/*v*). After centrifugation, the supernatant was used for UPLC-QTOF-MS analysis. The QC samples contained equal volumes of hippocampus homogenate and were prepared with the same procedures mentioned above. QC samples were used to evaluate the method repeatability, instrumental precision, and sample stability.

### 4.8. UPLC-QTOF-MS Conditions

Hippocampus metabolites were detected by the Acquity UPLC system coupled with Q-TOF mass spectrometer (Waters Corporation, Milford, MA, USA). Waters Acquity UPLC HSS T3 column (2.1 mm × 100 mm, 1.8 μm) was used for chromatography separation and column temperature was maintained at 40 °C. The mobile phases were composed of water (A) and ACN (B) both containing 0.1% FA, and the elution program was performed as follows, 1% B for 0–1 min, 1–25% B for 1–6 min, 25–80% for 6–10 min, 80–90% B for 10–12 min, 90–99% B for 12–16 min, and 1% B for 17–20 min. The flow rate was kept at 0.4 mL/min and the injection volume was 5 μL.

Both positive and negative ionization modes were used for MS analysis. The parameters of the mass spectrometer were optimized as follows: source temperature, 100 °C, de-solvation temperature, 400 °C, cone gas flow, 50 L/h, de-solvation gas flow, 600 L/h, capillary voltage, 3.0 kV for positive ionization mode and 2.5 kV for a negative ionization mode, and cone voltage, 40 V. Leucine-enkephalin was used as lock mass (m/z 556.2771 and 554.2615 in positive and negative ionization modes, respectively) and the data was acquired in the centroid mode with the mass range from m/z 50 to 1200. The QC samples were injected regularly (every eight samples) to monitor the stability of the LC/MS platform.

### 4.9. Data Preprocessing and Multivariate Statistical Analysis

The DataBridge software package was used to convert the raw data recorded by Masslynx 4.1 to the channel definition format (CDF) files. Data preprocessing included peak extraction and matching, nonlinear retention time alignment, and peak annotation, which were carried out by using R packages XCMS (version 3.4.1) and CAMERA (version 1.38.0) (Revolution Analytics, Redmond, WA, USA). The optimized parameters of XCMS and CAMERA were in agreement with our previous report [68]. The three-dimensional matrix, including sample names, peak index (retention time-m/z pairs), and peak intensities, was exported by XCMS into the Excel file. Additionally, the matrix was further progressed by SIMCA-P 13.0 software (Umetrics, Umea, Sweden). After mean-centering and pareto-scaling, unsupervised PCA and supervised PLS-DA were employed for multivariate statistical analysis. PCA could reflect an overview of the clustering trend and identify the outliers among the samples, while PLS-DA could maximize the separation among sample classes and find the biomarkers. A two-hundred-iteration permutation test was applied to test the fitting degree of the PLS-DA model.

### 4.10. Western Blot Assay

A Western blot assay was used to measure the expression of proteins associated with autophagy in the hippocampus of rats. The right hemisphere of the hippocampus was homogenized in ice-cold lysate buffer by an ultrasonic cell homogenizer, and then centrifuged at 13,000 rpm for 15 min at 4 °C. The supernatant was collected for quantification of protein concentration by a bicinchoninic acid (BCA) protein kit (Solarbio, Beijing, China). Equal amounts of protein (30 μg) in each sample were separated via SDS-PAGE gels (10% or 12%), which were transferred to the nitrocellulose (NC) membrane (Millipore, MA, USA). Subsequently, the NC membrane was blocked with 5% skim milk at room temperature for 90 min, and then incubated with specific primary antibodies, including anti-p-Akt (Ser473) (1:1000, Cell Signaling Technology (CST)), anti-Akt (1:1000, CST), anti-p-mTOR (Ser2448) (1:1000, CST), anti-mTOR (1:1000, CST), anti-p-ULK1 (Ser757) (1:1000, CST), anti-ULK1 (1:1000, CST), anti-p-AMPKα (Thr172) (1:800, CST), anti-AMPK (1:1000, CST), anti-p62 (1:1500, Proteintech), anti-Beclin 1 (1:1500, Proteintech), and anti-LC3 (1:1000, Sigma) overnight at 4 °C, which was followed by incubation with horseradish peroxidase (HRP)-conjugated secondary antibodies (1:5000, ABclonol) for 2 h at room temperature. The membrane was washed three times in 1 × TBS containing 0.2% Tween-20 (TBST) for 10 min. Afterward, the eECL developing reagent was added and the membrane was scanned by a ChemiDoc XRS system (Bio-Rad, Hercules, CA, USA). The gray values of protein bands were analyzed and expressed as the ratio of the target protein to an internal reference protein β-actin.

### 4.11. Statistical Analysis

All data are present as means ± standard error of the mean (SEM). A two-tailed *t*-test was performed to identify differential metabolites in the hippocampus between groups. SPSS 18.0 was used to evaluate the results derived from behavioral tests and Western blot with one-way analysis of variance (ANOVA), which was followed by post hoc Tukey’s test. *p* < 0.05 was considered statistically significant.

## Figures and Tables

**Table 1:** Identified biomarkers in the hippocampus of rats subjected to OBX and fluoxetine treatment.

No.	VIP ^a^	*m*/*z*	Retention Time (min)	Metabolites	Adduct ^b^	OBX/Sham	OBX + Flu/OBX	Pathways
1	1.44	90.05607	0.75	Sarcosine	M + H	↓ ***		Amino acid metabolism
2	1.42	106.0511	5.01	Serine	M + H	↓ ***	↓ #	Amino acid metabolism
3	1.31	148.0616	0.89	Glutamate	M + H	↓ **		Amino acid metabolism
4	1.33	158.0286	0.88	Homocysteine	M + Na	↓ **		Amino acid metabolism
5	1.78	398.3272	9.77	trans-Hexadec-2-enoyl carnitine	M + H	↑ **	↓ #	Fatty acid metabolism
6	2.3	400.343	10.12	Palmitoylcarnitine	M + H	↑ ***	↓ ##	Fatty acid metabolism
7	1.83	424.3427	9.93	Linoelaidyl carnitine	M + H	↑ *	↓ #	Fatty acid metabolism
8	1.71	426.3588	10.24	Vaccenyl carnitine	M + H	↑ *	↓ #	Fatty acid metabolism
9	3.9	151.0261	1.7	Xanthine	M-H	↑ *	↓ #	Purine metabolism
10	1.5	347.0355	0.93	Inosinic acid	M-H	↑ *	↓ ##	Purine metabolism
11	1.1	137.047	0.92	Hypoxanthine	M + H	↓ ***		Purine metabolism
12	1.44	132.078	0.75	Creatine	M + H	↓ ***	↓ #	Energy metabolism
13	1.11	119.037	0.91	Succinic acid	M + H	↓ *		Energy metabolism
14	1.27	112.0516	0.9	Cytosine	M + H	↓ ***		Others
15	1.28	214.0727	0.92	Droxidopa	M + H	↓ ***		Others
16	1.3	184.0746	10.5	Phosphocholine	M + H	↑ **		Others

^a^ VIP, variable importance in the projection, acquired from the PLS-DA model with a threshold of 1.0. ^b^ Adduct means the ionized form of a compound, which can be detected by UPLC-QTOF-MS in a positive or a negative ionization mode. Note, “↑”, up-regulation. “↓”, down-regulation. * *p* < 0.05, ** *p* < 0.01, *** *p* < 0.001 compared to the sham group. # *p* < 0.05, ## *p* < 0.01 compared to OBX + Flu group (*n* = 8 in each group). Flu, fluoxetine. OBX, olfactory bulbectomy.

## Supplementary Materials

Supplementary Materials can be found at https://www.mdpi.com/1422-0067/20/17/4282/s1.

## References

[1] Bromet E., Andrade L.H., Hwang I., Sampson N.A., Alonso J., de Girolamo G., de Graaf R., Demyttenaere K., Hu C., Iwata N. (2011). Cross-national epidemiology of DSM-IV major depressive episode. BMC Med..

[2] Zhang Y., Yuan S., Pu J., Yang L., Zhou X., Liu L., Jiang X., Zhang H., Teng T., Tian L. (2018). Integrated Metabolomics and Proteomics Analysis of Hippocampus in a Rat Model of Depression. Neuroscience.

[3] Aleksandrova I.Y., Kuvichkin V.V., Kashparov I.A., Medvinskaya N.I., Nesterova I.V., Lunin S.M., Samokhin A.N., Bobkova N.V. (2004). Increased level of beta-amyloid in the brain of bulbectomized mice. Biochemistry.

[4] Bobkova N., Vorobyov V., Medvinskaya N., Nesterova I., Tatarnikova O., Nekrasov P., Samokhin A., Deev A., Sengpiel F., Koroev D. (2016). Immunization Against Specific Fragments of Neurotrophin p75 Receptor Protects Forebrain Cholinergic Neurons in the Olfactory Bulbectomized Mice. J. Alzheimers Dis..

[5] Takahashi K., Nakagawasai O., Nemoto W., Odaira T., Sakuma W., Tan-No K. (2018). Antidepressant-like effect of aripiprazole via 5-HT1A, D1, and D2 receptors in the prefrontal cortex of olfactory bulbectomized mice. J. Pharmacol. Sci..

[6] Takahashi K., Nakagawasai O., Sugawara M., Sato A., Nemoto W., Tadano T., Tan-No K. (2018). Kappa Opioid Receptor Agonist Administration in Olfactory Bulbectomized Mice Restores Cognitive Impairment through Cholinergic Neuron Activation. Biol. Pharm. Bull..

[7] Zhou Y.F., Feng L., Liu X.M., Tao X., Wang L.S., Zhang M.D., Wang Z., Chen S.G., Chang Q. (2019). Urinary metabolic disturbance in the olfactory bulbectomized rats and the modulatory effects of fluoxetine. Life Sci..

[8] Ni Y., Su M., Lin J., Wang X., Qiu Y., Zhao A., Chen T., Jia W. (2008). Metabolic profiling reveals disorder of amino acid metabolism in four brain regions from a rat model of chronic unpredictable mild stress. FEBS Lett..

[9] Du H., Wang K., Su L., Zhao H., Gao S., Lin Q., Ma X., Zhu B., Dong X., Lou Z. (2016). Metabonomic identification of the effects of the Zhimu-Baihe saponins on a chronic unpredictable mild stress-induced rat model of depression. J. Pharm. Biomed. Anal..

[10] Shen L., Han B., Geng Y., Wang J., Wang Z., Wang M. (2017). Amelioration of cognitive impairments in APPswe/PS1dE9 mice is associated with metabolites alteration induced by total salvianolic acid. PLoS ONE.

[11] Morales-Medina J.C., Iannitti T., Freeman A., Caldwell H.K. (2017). The olfactory bulbectomized rat as a model of depression: The hippocampal pathway. Behav. Brain Res..

[12] Shehata M., Matsumura H., Okubo-Suzuki R., Ohkawa N., Inokuchi K. (2012). Neuronal stimulation induces autophagy in hippocampal neurons that is involved in AMPA receptor degradation after chemical long-term depression. J. Neurosci..

[13] Harris H., Rubinsztein D.C. (2011). Control of autophagy as a therapy for neurodegenerative disease. Nat. Rev. Neurol..

[14] Takacs-Vellai K., Bayci A., Vellai T. (2006). Autophagy in neuronal cell loss: A road to death. Bioessays.

[15] Nixon R.A., Yang D.S. (2012). Autophagy and neuronal cell death in neurological disorders. Cold Spring Harb. Perspect. Biol..

[16] Jia J., Le W. (2015). Molecular network of neuronal autophagy in the pathophysiology and treatment of depression. Neurosci. Bull..

[17] Kim D.H., Sarbassov D.D., Ali S.M., King J.E., Latek R.R., Erdjument-Bromage H., Tempst P., Sabatini D.M. (2002). mTOR interacts with raptor to form a nutrient-sensitive complex that signals to the cell growth machinery. Cell.

[18] Cleary C., Linde J.A., Hiscock K.M., Hadas I., Belmaker R.H., Agam G., Flaisher-Grinberg S., Einat H. (2008). Antidepressive-like effects of rapamycin in animal models: Implications for mTOR inhibition as a new target for treatment of affective disorders. Brain Res. Bull..

[19] Gassen N.C., Hartmann J., Zschocke J., Stepan J., Hafner K., Zellner A., Kirmeier T., Kollmannsberger L., Wagner K.V., Dedic N. (2014). Association of FKBP51 with priming of autophagy pathways and mediation of antidepressant treatment response: Evidence in cells, mice, and humans. PLoS Med..

[20] Alcocer-Gomez E., Casas-Barquero N., Williams M.R., Romero-Guillena S.L., Canadas-Lozano D., Bullon P., Sanchez-Alcazar J.A., Navarro-Pando J.M., Cordero M.D. (2017). Antidepressants induce autophagy dependent-NLRP3-inflammasome inhibition in Major depressive disorder. Pharmacol. Res..

[21] Deng W., Wang Y., Liu Z., Cheng H., Xue Y. (2014). HemI: A toolkit for illustrating heatmaps. PLoS ONE.

[22] Peitl M.V., Proloscic J., Blazevic-Zelic S., Skarpa-Usmiani I., Peitl V. (2011). Symptoms of agitated depression and/or akathisia. Psychiatr. Danub..

[23] Borre Y.E., Panagaki T., Koelink P.J., Morgan M.E., Hendriksen H., Garssen J., Kraneveld A.D., Olivier B., Oosting R.S. (2014). Neuroprotective and cognitive enhancing effects of a multi-targeted food intervention in an animal model of neurodegeneration and depression. Neuropharmacology.

[24] Bobkova N.V., Novikov V.V., Medvinskaya N.I., Aleksandrova I.Y., Nesterova I.V., Fesenko E.E. (2018). Effect of weak combined static and extremely low-frequency alternating magnetic fields on spatial memory and brain amyloid-beta in two animal models of Alzheimer’s disease. Electromagn. Biol. Med..

[25] Machado D.G., Cunha M.P., Neis V.B., Balen G.O., Colla A.R., Grando J., Brocardo P.S., Bettio L.E., Dalmarco J.B., Rial D. (2012). Rosmarinus officinalis L. hydroalcoholic extract, similar to fluoxetine, reverses depressive-like behavior without altering learning deficit in olfactory bulbectomized mice. J. Ethnopharmacol..

[26] Hitti F.L., Siegelbaum S.A. (2014). The hippocampal CA2 region is essential for social memory. Nature.

[27] Mahar I., Bambico F.R., Mechawar N., Nobrega J.N. (2014). Stress, serotonin, and hippocampal neurogenesis in relation to depression and antidepressant effects. Neurosci. Biobehav. Rev..

[28] Morales-Medina J.C., Juarez I., Venancio-Garcia E., Cabrera S.N., Menard C., Yu W., Flores G., Mechawar N., Quirion R. (2013). Impaired structural hippocampal plasticity is associated with emotional and memory deficits in the olfactory bulbectomized rat. Neuroscience.

[29] Jaako-Movits K., Zharkovsky T., Pedersen M., Zharkovsky A. (2006). Decreased hippocampal neurogenesis following olfactory bulbectomy is reversed by repeated citalopram administration. Cell Mol. Neurobiol..

[30] Moriguchi S., Han F., Nakagawasai O., Tadano T., Fukunaga K. (2006). Decreased calcium/calmodulin-dependent protein kinase II and protein kinase C activities mediate impairment of hippocampal long-term potentiation in the olfactory bulbectomized mice. J. Neurochem..

[31] Rinwa P., Kumar A. (2013). Quercetin suppress microglial neuroinflammatory response and induce antidepressent-like effect in olfactory bulbectomized rats. Neuroscience.

[32] Borre Y., Sir V., de Kivit S., Westphal K.G., Olivier B., Oosting R.S. (2012). Minocycline restores spatial but not fear memory in olfactory bulbectomized rats. Eur. J. Pharmacol..

[33] Zheng P., Gao H.C., Li Q., Shao W.H., Zhang M.L., Cheng K., Yang D.Y., Fan S.H., Chen L., Fang L. (2012). Plasma metabonomics as a novel diagnostic approach for major depressive disorder. J. Proteome Res..

[34] Zhou X., Liu L., Zhang Y., Pu J., Yang L., Zhou C., Yuan S., Zhang H., Xie P. (2017). Metabolomics identifies perturbations in amino acid metabolism in the prefrontal cortex of the learned helplessness rat model of depression. Neuroscience.

[35] Chen G., Yang D., Yang Y., Li J., Cheng K., Tang G., Zhang R., Zhou J., Li W., Liu Z. (2015). Amino acid metabolic dysfunction revealed in the prefrontal cortex of a rat model of depression. Behav. Brain Res..

[36] Pham T.H., Gardier A.M. (2019). Fast-acting antidepressant activity of ketamine: highlights on brain serotonin, glutamate, and GABA neurotransmission in preclinical studies. Pharmacol. Ther..

[37] Otte D.M., Barcena de Arellano M.L., Bilkei-Gorzo A., Albayram O., Imbeault S., Jeung H., Alferink J., Zimmer A. (2013). Effects of chronic D-serine elevation on animal models of depression and anxiety-related behavior. PLoS ONE.

[38] Billard J.M. (2008). D-serine signalling as a prominent determinant of neuronal-glial dialogue in the healthy and diseased brain. J. Cell Mol. Med..

[39] Malkesman O., Austin D.R., Tragon T., Wang G., Rompala G., Hamidi A.B., Cui Z., Young W.S., Nakazawa K., Zarate C.A. (2012). Acute D-serine treatment produces antidepressant-like effects in rodents. Int. J. Neuropsychopharmacol..

[40] Nagasawa M., Otsuka T., Togo Y., Yamanaga M., Yoshida J., Uotsu N., Teramoto S., Yasuo S., Furuse M. (2017). Single and chronic L-serine treatments exert antidepressant-like effects in rats possibly by different means. Amino Acids.

[41] Huang C.C., Wei I.H., Huang C.L., Chen K.T., Tsai M.H., Tsai P., Tun R., Huang K.H., Chang Y.C., Lane H.Y. (2013). Inhibition of glycine transporter-I as a novel mechanism for the treatment of depression. Biol. Psychiatry.

[42] Almeida O.P., Lautenschlager N., Flicker L., Leedman P., Vasikaran S., Gelavis A., Ludlow J. (2004). Association between homocysteine, depression, and cognitive function in community-dwelling older women from Australia. J. Am. Geriatr. Soc..

[43] Ford A.H., Almeida O.P. (2012). Effect of homocysteine lowering treatment on cognitive function: A systematic review and meta-analysis of randomized controlled trials. J. Alzheimers Dis..

[44] Bottiglieri T., Laundy M., Crellin R., Toone B.K., Carney M.W., Reynolds E.H. (2000). Homocysteine, folate, methylation, and monoamine metabolism in depression. J. Neurol. Neurosurg. Psychiatry.

[45] Pazini F.L., Cunha M.P., Rodrigues A.L.S. (2019). The possible beneficial effects of creatine for the management of depression. Prog. Neuropsychopharmacol. Biol. Psychiatry.

[46] Joncquel-Chevalier Curt M., Voicu P.M., Fontaine M., Dessein A.F., Porchet N., Mention-Mulliez K., Dobbelaere D., Soto-Ares G., Cheillan D., Vamecq J. (2015). Creatine biosynthesis and transport in health and disease. Biochimie.

[47] Kim S.Y., Lee Y.J., Kim H., Lee D.W., Woo D.C., Choi C.B., Chae J.H., Choe B.Y. (2010). Desipramine attenuates forced swim test-induced behavioral and neurochemical alterations in mice: An in vivo(1)H-MRS study at 9.4T. Brain Res..

[48] Knox D., Perrine S.A., George S.A., Galloway M.P., Liberzon I. (2010). Single prolonged stress decreases glutamate, glutamine, and creatine concentrations in the rat medial prefrontal cortex. Neurosci. Lett..

[49] Michael-Titus A.T., Albert M., Michael G.J., Michaelis T., Watanabe T., Frahm J., Pudovkina O., van der Hart M.G., Hesselink M.B., Fuchs E. (2008). SONU20176289, a compound combining partial dopamine D(2) receptor agonism with specific serotonin reuptake inhibitor activity, affects neuroplasticity in an animal model for depression. Eur. J. Pharmacol..

[50] Soder J., Wernersson S., Dicksved J., Hagman R., Ostman J.R., Moazzami A.A., Hoglund K. (2019). Indication of metabolic inflexibility to food intake in spontaneously overweight Labrador Retriever dogs. BMC Vet. Res..

[51] Mutomba M.C., Yuan H., Konyavko M., Adachi S., Yokoyama C.B., Esser V., McGarry J.D., Babior B.M., Gottlieb R.A. (2000). Regulation of the activity of caspases by L-carnitine and palmitoylcarnitine. FEBS Lett..

[52] Yu H.Y., Yin Z.J., Yang S.J., Ma S.P., Qu R. (2016). Baicalin Reverses Depressive-Like Behaviours and Regulates Apoptotic Signalling Induced by Olfactory Bulbectomy. Phytother. Res..

[53] Zhou X., Liu L., Lan X., Cohen D., Zhang Y., Ravindran A.V., Yuan S., Zheng P., Coghill D., Yang L. (2018). Polyunsaturated fatty acids metabolism, purine metabolism and inosine as potential independent diagnostic biomarkers for major depressive disorder in children and adolescents. Mol. Psychiatry.

[54] Garcia-Esparcia P., Hernandez-Ortega K., Ansoleaga B., Carmona M., Ferrer I. (2015). Purine metabolism gene deregulation in Parkinson’s disease. Neuropathol. Appl. Neurobiol..

[55] Jung C.H., Ro S.H., Cao J., Otto N.M., Kim D.H. (2010). mTOR regulation of autophagy. FEBS Lett..

[56] Alers S., Loffler A.S., Wesselborg S., Stork B. (2012). Role of AMPK-mTOR-Ulk1/2 in the regulation of autophagy: Cross talk, shortcuts, and feedbacks. Mol. Cell. Biol..

[57] Wang Z., Wilson W.A., Fujino M.A., Roach P.J. (2001). Antagonistic controls of autophagy and glycogen accumulation by Snf1p, the yeast homolog of AMP-activated protein kinase, and the cyclin-dependent kinase Pho85p. Mol. Cell. Biol..

[58] Huang Z., Huang X., Wang Q., Jiang R., Sun G., Xu Y., Wu Q. (2018). Extract of Euryale ferox Salisb exerts antidepressant effects and regulates autophagy through the adenosine monophosphate-activated protein kinase-UNC-51-like kinase 1 pathway. IUBMB Life.

[59] Huang X., Wu H., Jiang R., Sun G., Shen J., Ma M., Ma C., Zhang S., Huang Z., Wu Q. (2018). The antidepressant effects of a-tocopherol are related to activation of autophagy via the AMPK/mTOR pathway. Eur. J. Pharmacol..

[60] Hu J., Huang H.Z., Wang X., Xie A.J., Wang X., Liu D., Wang J.Z., Zhu L.Q. (2015). Activation of Glycogen Synthase Kinase-3 Mediates the Olfactory Deficit-Induced Hippocampal Impairments. Mol. Neurobiol..

[61] Jimenez-Sanchez L., Linge R., Campa L., Valdizan E.M., Pazos A., Diaz A., Adell A. (2016). Behavioral, neurochemical and molecular changes after acute deep brain stimulation of the infralimbic prefrontal cortex. Neuropharmacology.

[62] Kelly J.P., Wrynn A.S., Leonard B.E. (1997). The olfactory bulbectomized rat as a model of depression: An update. Pharmacol. Ther..

[63] Jindal A., Mahesh R., Bhatt S. (2015). Type 4 phosphodiesterase enzyme inhibitor, rolipram rescues behavioral deficits in olfactory bulbectomy models of depression: Involvement of hypothalamic-pituitary-adrenal axis, cAMP signaling aspects and antioxidant defense system. Pharmacol. Biochem. Behav..

[64] Shibata S., Nakanishi H., Watanabe S., Ueki S. (1984). Effects of chronic administration of antidepressants on mouse-killing behavior (muricide) in olfactory bulbectomized rats. Pharmacol. Biochem. Behav..

[65] Gotoh L., Saitoh A., Yamada M., Fujii H., Nagase H., Yamada M. (2017). Effects of repeated treatment with a delta opioid receptor agonist KNT-127 on hyperemotionality in olfactory-bulbectomized rats. Behav. Brain Res..

[66] Morris R. (1984). Developments of a water-maze procedure for studying spatial learning in the rat. J. Neurosci. Methods.

[67] Holubova K., Kleteckova L., Skurlova M., Ricny J., Stuchlik A., Vales K. (2016). Rapamycin blocks the antidepressant effect of ketamine in task-dependent manner. Psychopharmacology.

[68] Feng L., Yue X.F., Chen Y.X., Liu X.M., Wang L.S., Cao F.R., Wang Q., Liao Y.H., Pan R.L., Chang Q. (2016). LC/MS-based metabolomics strategy to assess the amelioration effects of ginseng total saponins on memory deficiency induced by simulated microgravity. J. Pharm. Biomed. Anal..

