# Supplementary material for: Hippocampus Metabolic Disturbance and Autophagy Deficiency in Olfactory Bulbectomized Rats and the Modulatory Effect of Fluoxetine
Source: Int J Mol Sci. 2019 Sep 1;20(17):4282. doi: 10.3390/ijms20174282 (PMC6747550; doi:10.3390/ijms20174282)
Supplement: Supplementary file 1 [file ijms-20-04282-s001.pdf]

Supplementary materials

Table S1 Repeatability, precision and 4 °C stability of hippocampus QC samples in positive and negative ionization modes of UPLC-QTOF-MS data.

**RT_*m/z***	**Repeatability (RSD, %)**		**Precision (RSD, %)**		**4 °C stability (RSD, %)**	
	**RT**	**Peak Area**	**RT**	**Peak Area**	**RT**	**Peak Area**
Hippocampus sample in positive ionization mode						
0.90_102.0599	0.66	12.59	0.36	11.75	0.39	1.76
2.78_137.047	0.00	5.42	0.17	4.81	0.02	9.35
3.20_120.0818	0.09	10.62	0.06	9.98	0.22	4.56
5.01_621.0631	0.06	3.57	0.01	2.77	0.05	8.63
6.97_714.7231	0.04	4.09	0.00	3.43	0.07	8.12
8.26_820.6949	0.01	4.30	0.08	4.43	0.15	6.19
Hippocampus sample in negative ionization mode						
0.89_329.0179	0.65	12.86	0.00	13.88	2.35	9.31
3.52_218.1045	0.18	9.48	0.29	13.64	0.09	13.31
5.22_255.0907	0.23	12.40	0.09	9.85	0.05	11.84
8.31_242.1789	0.14	8.78	0.10	12.90	0.04	2.15
10.39_541.3306	0.06	9.66	0.08	12.13	0.03	8.11
12.41_259.2411	0.04	12.27	0.03	8.26	0.04	4.25

RT: Retention time; RSD: Relative standard deviation
